# Annotations

**Published:** 1901-11-23

**Authors:** 


### Annotations.

### Fractured Ribs in the Insane.

There is, perhaps, no asylum in England in which a coroner's jury has not sat and deliberated on some so-called mystery connected with the death of a patient in whose case the post-mortem examination has revealed fractured ribs; and the public, judging the case from the greatly epitomised accounts which appear in the newspapers, and the not always too friendly comments of the editor, comes to the conclusion that the fractures were caused by unkind treatment. That such fractures are sometimes the result of rough usage or of struggles, avoidable or unavoidable, is probably true, but these cases are quite exceptional. It is beyond controversy that an extreme brittleness is occasionally met with in general paralysis of the insane and in some other forms of organic dementia; and in advanced cases it would be difficult even to clean or attend to the patient without the risk of fracturing a rib. Some of these patients will resent the slightest touch, others are in the highest degree restless, and in others the reflexes are so deadened that when a fracture has occurred it is not the simple matter to diagnose it that it would be in the case of a sane individual. These fractures have been seen in patients who had not been out of bed for weeks, and who had never been in any way roughly handled. Occasionally, the post-mortem examination shows that the ribs have reached such a degree of brittleness that a piece two inches long may easily be broken between the finger and thumb. This is a condition which has been demonstrated to a coroner's jury. The mystery in these extreme cases is not so much the existence of fractured ribs as the presence of unfractured ones. It sometimes happens that the fracture is almost exactly transverse. There is no displacement, no external mark, no complaint by the patient, no sensation of pain if the part be touched; and the post-mortem examination may show that little or no attempt at union has taken place. Can it be wondered at that these cases are not always diagnosed before death? We are far from holding that the bones of all lunatics are more easily broken than those of the sane. Such a statement would be nonsense. Nor have we any wish to write anything which might be construed into an attempt to excuse any asylum official who may carelessly examine a patient, or who may have ill-treated a patient; but no asylum should have a black mark tacked to its name merely because of some injury, undetected during life, from which an inmate may have suffered: an injury which the most skilful might have overlooked.

### The Policy of the Tea-kettle.

In a lecture recently delivered at the United Service Institution on "Typhoid, the Destroyer of Armies," Dr. Leigh Canney certainly advanced the subject a stage by putting into actual figures the degree to which the impedimenta of an army in the field would be increased by taking with it the means of supplying the soldiers with boiled water. In many of his details he may require correction, and it is possible that in some cases his plan may be found impracticable in the face of military requirements, but we can have no doubt that so far as fixed posts and more or less stationary camps are concerned and they are the great foci of typhoid fever, the policy of the tea kettle, that is of boiling every drop of water that is to be drunk, is the right one. The great importance of Dr. Canney's lecture lay in the fact that it laid down a complete scheme, and showed what a very small addition to the transport would be required to eliminate the main cause by which typhoid is caused. He pointed out that the general transport for the supply of an army of 200,000 is about 2,000 tons per day, while the fuel required to ensure to each man four pints of boiled water per day would be about five tons. In other words that by increasing the baggage of an army by one four-hundredth part, the whole of the water drunk by that army could be boiled. Such an addition to the transport of an army would be as nothing compared with that which is involved by even a small outbreak of typhoid fever. If, however, such an outbreak is to be prevented by the means proposed, it is essential that the whole business must be worked on some such an organised system as that which is proposed by Dr. Canney. Half measures can only bring discredit on the policy of the tea-kettle.

### The Status of Medical Herbalists.

At a time when it is in the highest degree necessary to maintain a high standard of medical skill, any judicial recognition of irregular practice is very prejudicial to the interests of the public health. The recent decision of his Honour Judge French is therefore extremely regrettable. The learned judge has, in effect, ruled that the so-called medical herbalists are protected by an old Act of Henry VIII. in prescribing their specifics. The view taken by the Apothecaries' Society, who were the plaintiffs in a penal action against a London herbalist, is that although still on the Statute Book this Act is really obsolete, and that under the Apothecaries Act, if not under the Medical Acts, any acting or practising as a doctor, unless by a person duly qualified according to the requirements of those Acts, is illegal. That this is in accordance with the spirit of all modern legislation on the subject can scarcely be gainsaid. The very wide terms in which the Apothecaries Act is framed raises an almost irresistible presumption that a mere saving clause contained in one of the earliest enactments dealing with the practice of medicine and surgery is not to override the substantive provisions of later legislation. Under the Apothecaries Act it has been held that even the pharmaceutical chemist who has passed a thorough examination in materia medica, chemistry, and physics may not prescribe the simplest remedies for his customers (see Apothecaries' Company *v.* Nottingham, 1876, 34 *Law Times Reports*, p. 76); and it is difficult to see why a herbalist who has no recognised legal qualification—whatever may be the virtue of a certificate from the National Association of Medical Herbalists of Great Britain—should be in any more favoured position. Our medical laws are surely in an anomalous condition when anyone who chooses to label himself with a trade description which carries with it no warranty of quality can freely compete with those who are compelled by law to pass a long and expensive training before they are allowed to embark on perhaps the most arduous and delicate of human callings.

